# Risk factors and prognosis of orotracheal intubation in aquaporin-4-IgG neuromyelitis optica spectrum disorder attacks

**DOI:** 10.1186/s13613-023-01213-x

**Published:** 2024-01-08

**Authors:** Edouard Januel, Vincent Brochard, Loïc Le Guennec, Elisabeth Maillart, Céline Louapre, Catherine Lubetzki, Nicolas Weiss, Sophie Demeret, Caroline Papeix

**Affiliations:** 1 Neurology Department, Centre de Référence des Maladies Inflammatoires Rares du Cerveau et de la Moelle (MIRCEM), Pitié-Salpêtrière University Hospital, AP-HP, Paris, France; 2 INSERM, Institut Pierre Louis d’Epidémiologie et de Santé Publique, Hôpital Pitié Salpêtrière, AP-HP, Sorbonne Université, 47-83 Bd de l’Hôpital, Paris, France; 3 unité de Médecine Intensive Réanimation à orientation Neurologique, Département de Neurologie, Hôpital de la Pitié-Salpêtrière, AP-HP, Sorbonne Université, 47-83 boulevard de l’Hôpital, 75013 Paris, France; 4 Groupe de Recherche Clinique en REanimation et Soins Intensifs du Patient en Insuffisance Respiratoire aiguE (GRC-RESPIRE) Sorbonne Université, Paris, France; 5 Brain Liver Pitié-Salpêtrière (BLIPS) Study Group, INSERM UMR_S 938, Centre de Recherche Saint-Antoine, Maladies métaboliquesbiliaires et fibro-inflammatoire du foie, Institute of Cardiometabolism and Nutrition (ICAN), Paris, France; 6 Neurology Department, Fondation A. de Rothshchild Hospital, Paris, France

## Abstract

**Background:**

Aquaporin-4 immunoglobulin G Neuro Myelitis Optica spectrum disorders attacks (NMOSD-AQP4-IgG+ attacks) can cause respiratory failure requiring orotracheal intubation (OTI), but the risk factors and outcomes of OTI during attacks remain unclear. Our primary objective was to identify the clinical and radiological risk factors for OTI in NMOSD-AQP4-IgG+ attacks. As a secondary objective, we aimed to evaluate the prognosis of OTI-attacks.

**Methods:**

We retrospectively analyzed NMOSD-AQP4-IgG+ attacks at the Pitié-Salpêtrière Hospital (Jan 2010–Jan 2021), excluding isolated optic neuritis. The primary outcome was the need for OTI due to neurological dysfunction an attack (OTI-attack). The secondary outcome was attack’s poor recovery after 12 months, defined as a modified Rankin score (mRS) > 2 in patients with an initial mRS ≤ 2, or an increase ≥ 1 point in mRS in other patients. Analyses were performed using a binomial generalized linear mixed model, with a random intercept for the patient ID to account for within-patient correlations.

**Results:**

Seventy-three attacks in 44 patients NMOSD-AQP4-IgG+ were analyzed. Of 73 attacks, 8 (11%) required OTI during the attack, related to acute restrictive respiratory failure (*n* = 7) and/or severe swallowing disorder (*n* = 2). None of the OTI-attacks occurred in patients previously treated with active disease-modifying treatment (DMT), while 36 (55.4%) of the non-OTI-attacks occurred in patients who were already on active DMT. On admission, OTI-attacks were more likely to have upper limbs motor paresis of (75.0% versus 29.2%, *p* = 0.366) and dyspnea (3 [50.0%] versus 4 [6.6%], *p* = 0.002) compared to non-OTI-attacks. MRI analysis showed that OTI-attacks had edematous lesions in the cervical spinal cord, mainly at levels C1 (75% versus 0% in non-OTI-attacks), C2 (75% versus 1.9%), C3 (62.5% versus 1.9%), and C4 and C5 levels (50% versus to 3.9%). One OTI-attack resulted in the death of one patient. Five patients with OTI-attack had mRS ≤ 2 one year after OTI-attack. Two (25%) OTI-attacks had poor recovery compared to 15 (24.2%) non-OTI-attacks (*p* = 0.468).

**Conclusion:**

OTI-attacks occurred in untreated NMOSD-AQP4-IgG+ patients and were associated with edematous upper cervical lesions. The prognosis of these attacks may be favorable, and warrant maximal medical and supportive treatment.

*Trial registration* This was a retrospective observational monocentric cohort study nested in the NOMADMUS cohort (ClinicalTrials.gov Identifier: NCT02850705)

**Graphical Abstract:**

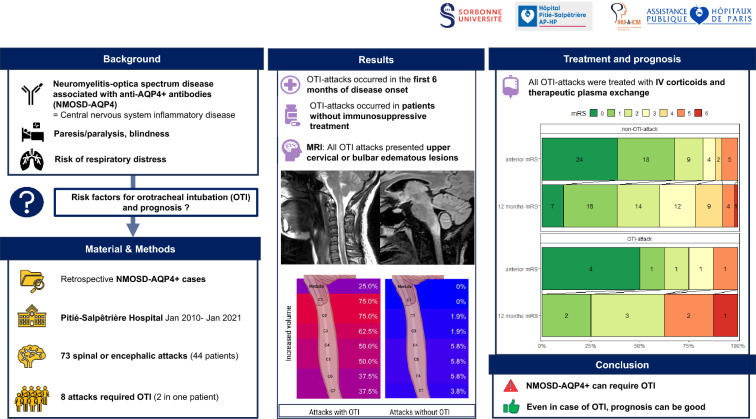

**Supplementary Information:**

The online version contains supplementary material available at 10.1186/s13613-023-01213-x.

## Introduction

Neuro Myelitis Optica spectrum disorders (NMOSD) are a rare auto-immune group of disorders affecting the central nervous system (CNS). Initially described as diseases affecting exclusively optic nerves and spinal cord, the clinical spectrum has been broadened after the identification of aquaporin-4 immunoglobulin G (AQP4-IgG) [1–4]. In addition to these two elective locations, the core clinical characteristics of the International Panel for NMOSD Diagnosis (IPND) criteria, included also area postrema, brainstem, diencephalon, and hemispheric locations [5]. In the heterogeneous group of NMOSD, which includes anti-AQP4 IgG+ cases, myelin oligodendrocyte glycoprotein antibody-associated disease (MOGAD), and seronegative NMOSD, NMOSD-AQP4-IgG+ attacks are notably severe and result in long-term disability [6–8].

NMOSD-AQP4-IgG+ attacks can be life-threatening, trigger respiratory failure requiring oro-tracheal intubation (OTI) in intensive care unit (ICU), and lead to severe disability. In the absence of immunosuppressive treatment, the issue is fatal in one-third of relapsing NMOSD patient, because of respiratory failure [9]. NMOSD-AQP4-IgG+ attacks are now managed with intensive medical treatment at the acute stage, including high-dose intravenous corticosteroids and therapeutic plasma exchange for the most severe attacks [10].

The clinical and radiological risk factors of OTI in NMOSD-AQP4-IgG+ attacks, as well as their prognosis in the aera of active treatment remain unknown. Our first objective was to determine the clinical and radiological factors associated with the risk of OTI during NMOSD-AQP4-IgG+ attacks. Our secondary objectives were to assess the prognosis of OTI-attacks compared with non-OTI-attacks, and to determine whether OTI is a risk factor for poor attack recovery, and to evaluate the clinical characteristics of NMOSD-AQP4-IgG+ attacks requiring ICU admission.

## Materials and methods

This was a retrospective observational monocentric cohort study nested in the NOMADMUS cohort (ClinicalTrials.gov Identifier: NCT02850705).

The primary outcome was the need for OTI due to neurological dysfunction during an NMOSD-AQP4-IgG+ attack. Attacks that were managed with non-invasive ventilation alone were classified as non-OTI-attacks. The secondary outcomes were (1) adverse outcome at 12 months after attack onset, defined as modified Rankin Score (mRS) > 2 for patients with initial mRS ≤ 2, or an increase ≥ 1 point in mRS for patients with initial mRS > 2; and (2) admission in intensive care unit (ICU) during the attack.

We included all consecutive attacks of NMOSD-AQP4-IgG+ patients fulfilling IPND-criteria [5] hospitalized at Pitié-Salpêtrière hospital from January 2010 to January 2021. All patients were aged > 18 years. NMOSD-AQP4-IgG+ attacks were defined as occurrence, recurrence or worsening of neurological symptoms lasting at least 24 h. Similar symptoms occurring within a month were considered as part of the same attack. NMOSD-AQP4-IgG+ attacks with isolated optic neuritis were not included in this study.

Were recorded age at disease onset, birthplace (categorized as “Metropolitan French area”; “West indies—Guyana French area”; “Asia”; “North Africa”; “Sub-Saharan Africa”; “Middle East”), age and disease duration at attack onset, active NMOSD-AQP4-IgG+ disease modifying therapy (DMT) at the hospital admission (DMT was defined as active if it has been received for at least 3 months), neurological exam, expanded disability severity score (EDSS) [11] at admission, 3 months 6 months and 12 months after attack, treatment of the attack (IV pulsed corticosteroids, therapeutic plasma exchange [TPE]). For attacks hospitalized in ICU were recorded the reason for ICU admission, and the Simplified Acute Physiology Score II (SAPS II). For OTI-attacks, were also recorded: reason for OTI, duration of mechanical ventilation, occurrence of aspiration or ventilator-associated pneumonia, need for tracheostomy. The cumulative duration of invasive mechanical ventilation included the period of orotracheal intubation, as well as the duration of mechanical ventilation via tracheostomy. This period ended when the patient was weaned from permanent mechanical ventilation, irrespective of the presence of a tracheostomy. Radiological characteristics were recorded: new lesion on T2 weighted sequences, gadolinium enhancement on T1 weighted sequences in brain and spinal MRI. We also recorded the presence of increased volume (i.e., oedema) in the cervical spinal cord and medulla oblongata. All MRI analyses were based on neuroradiological reports and two neurologists’ reviews.

### Statistical analysis

Categorical variables were expressed as number (%) and continuous variables as median, 25th percentile (P25) and 75th percentile (P75). All analyses were conducted using a binomial generalized linear mixed model, with a random intercept for patient ID to account for within patient correlation. In cases of complete separation (i.e., if an outcome variable completely segregated a predictor variable), *p*-values were not computed. We assessed risk factors associated with attack poor recovery using a binomial generalized linear mixed model, which included a random intercept for patient identification. Variables included in the multivariable analysis were OTI and variables associated with poor attack recovery in the univariate analysis. *P*-values below 0.05 defined statistical significance. In case of missing data, no imputation was carried out. All analyses were made on R 4.1.1, and figures were generated using Rstudio and Biorender.

### Standard protocol approvals, registrations, and patient consents

NOMADMUS cohort gathers data on patients with NMOSD collected by all French expert NMOSD centers and NOMADMUS network, using routinely European Database for Multiple Sclerosis (EDMUS) software as a medical file for all their NMOSD patients [12]. Patients enrolled in the NOMADMUS registry provided their written consent for participation. This registry was approved by a French ethical committee (Comité de Protection des Personnes [CPP]: reference 2019-Ă6-51). As the NOMADMUS cohort does not routinely collect information on need for ICU or OTI use during an attack, these supplementary data were collected retrospectively from medical files. This study was recorded in the registry of processing operations of Assistance Publique—Hôpitaux de Paris (No. 20210419181607), in accordance with the Commission Nationale de l'Informatique et des Libertés (CNIL) [13].

## Results

Seventy-three spinal or encephalic attacks from 44 NMOSD-AQP4-IgG+ patients were included between January 2010 and January 2021.

### Clinical characteristics of OTI versus non-OTI-attacks

Eight out of 73 attacks (11% of all attacks), required OTI because of a CNS dysfunction. The results are summarized in Table 1. Reason for OTI was acute restrictive respiratory failure (*n* = 7) and/or severe swallowing disorder (*n* = 2). None of OTI-attacks occurred while the patient was treated with active DMT, while 36 (55.4%) of the non-OTI-attacks occurred in patients who were already on active DMT. All but one OTI-attacks (87.5%) occurred in the first 6 months after disease onset, compared with 32.8% (*n* = 21) for non-OTI-attack (*p* = 0.411). At admission, OTI-attacks had higher EDSS score (median [IQR] EDSS 7.5 [6; 8.5] for OTI-attacks versus 6 [3.5;8] for non-OTI-attacks, *p* = 0.029), reported more frequent dyspnea (3 [50%] versus 4 [6.6%], *p* = 0.002), and 75% of OTI-attacks had upper limbs motor paresis (versus 29.2% of non-OTI-attacks, *p* = 0.366).

**Table 1:** Clinical characteristics of attacks requiring orotracheal intubation versus attacks not requiring orotracheal intubation

	All attacks, *N* = 73	OTI-attacks, *N* = 8 (11.0%)	Non-OTI-attacks, *N* = 65 (89.0%)	*P*-value
Gender: female/male	64 (87.7)	7 (87.5)	57 (87.7)	0.925
Place of birth				–
France—metropolitan area	34 (46.6)	1 (12.5)	33 (50.8)	
France–West indies–Guyana	6 (8.2)	4 (50.0)	2 (3.1)	
Asia	6 (8.2)	0 (0.0)	6 (9.2)	
North Africa	6 (8.2)	0 (0.0)	6 (9.2)	
Sub-Saharan Africa	20 (27.4)	2 (25.0)	18 (27.7)	
Middle East	1 (1.4)	1 (12.5)	0 (0.0)	
Age at start of the disease, years, median (IQR)	33.20 [21.30, 46.90]	47.15 [33.18, 55.70]	32.90 [19.60, 43.40]	0.662
Age at attack onset, years	43 (31–53)	50 (42–56)	43 (28–53)	0.834
First attack of NMOSD-AQP4 disease	22 (30.1)	4 (50)	18 (27.7)	0.592
Attack occurring in the first 6 months of NMOSD-AQP4 disease	28 (38.9)	7 (87.5)	21 (32.8)	0.411
Any active NMOSD disease modifying therapy (DMT)^a^	36 (49.3)	0 (0)	36 (55.4)	–
Type of DMT				
Azathioprine	8 (11)	–	8 (12.3)	
Mycophenolate mofetil	11 (15.1)	–	11 (16.9)	
Rituximab	8 (11)	–	8 (12.3)	
Cyclophosphamide	3 (4.1)	–	3 (4.6)	
Methotrexate	4 (5.5)	–	4 (6.2)	
TPE	6 (8.2)	–	6 (9.2)	
EDSS before attack	2.0 (0–3.5)	1.0 (0–3.5)	2.0 (0–3.5)	0.460
Modified Rankin scale before attack	1 (0–2)	0.5 (0–3.25)	1 (0–2)	0.380
Clinical examination at the hospital entry				
EDSS at admission	6.0 (3.5–8)	7.5 (6.0–8.5)	6.0 (3.5–8)	0.029
Lower limb paresis	46 (63)	6 (75)	40 (61.5)	0.460
Upper limb paresis	25 (34.2)	6 (75)	19 (29.2)	0.366
Dyspnea (number of patients with available data)	7 (9.7) (71)	3 (50) (6)	4 (6.6) (65)	0.002

Results are expressed in median (interquartile range) or *N* (%) unless otherwise specified

*NA* number of observations non available, *OTI* orotracheal intubation, *DMT* disease modifying therapy

*P*-value is estimated with binomial generalized linear mixed model, with a random intercept for patient ID. Due to separation issues, *p*-value has not been estimated for some variables

^a^Active DMT was defined if it has been received for at least 3 months before the attack first symptoms

Two attacks from a single patient required exclusive non-invasive ventilation for durations of 7 and 3 days, respectively, without the need for OTI; these two episodes were classified as non-OTI-attacks. The patient had a long history of recurrent AQP4 attacks spanning 18 years, with more than 15 attacks since disease onset, and already had chronic severe tetraparesis.

### Radiological analysis of OTI versus non-OTI-attacks

**Fig. 1:**
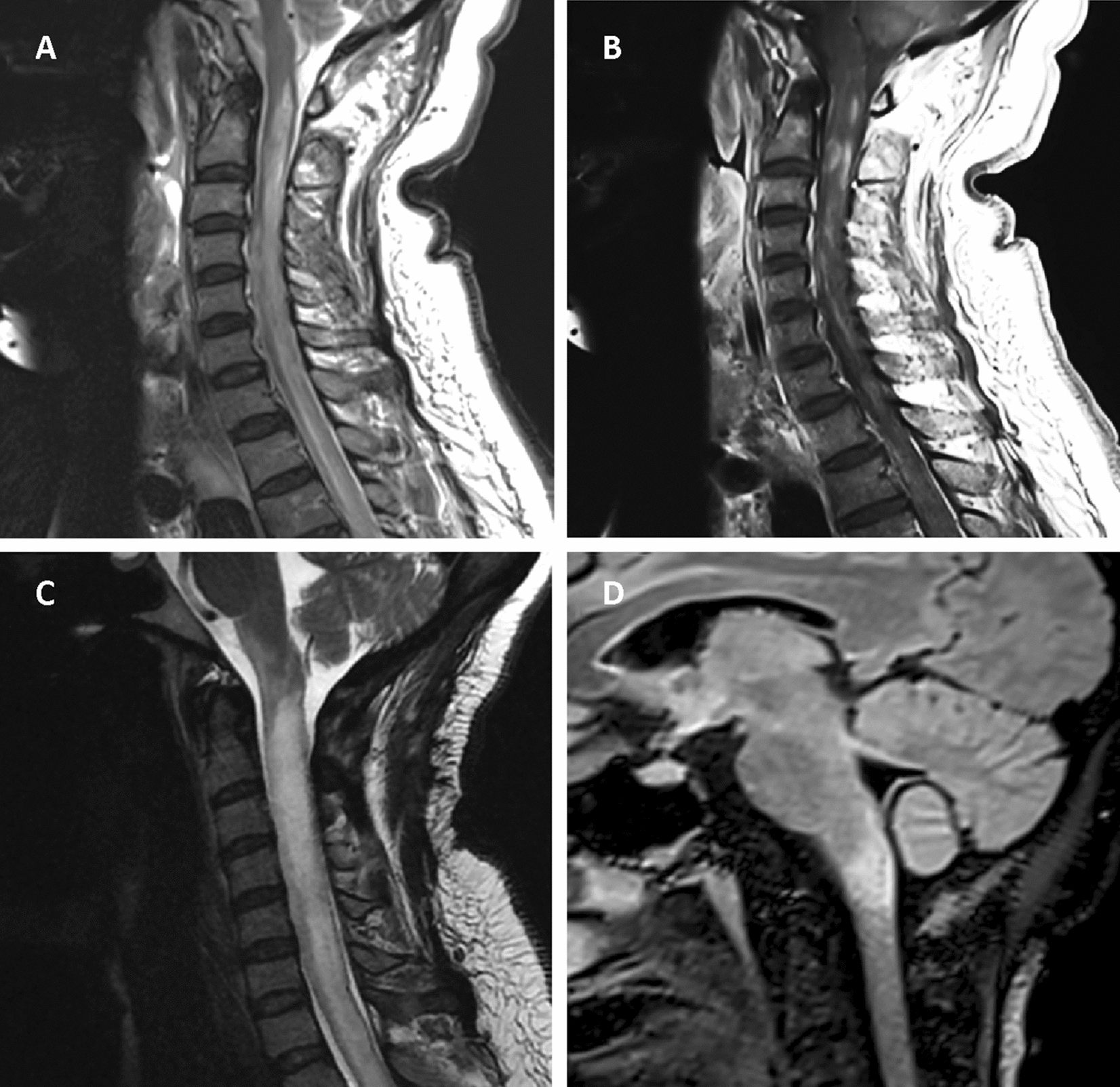
MRI features of NMOSD-AQP4+ patients with OTI-attacks. **A**, **B** Are derived from the same patient. **A**, **C** Are sagittal spinal cord T2 MRI. **B** Is sagittal T1 with gadolinium. **D** Is FLAIR MRI

**Fig. 2:**
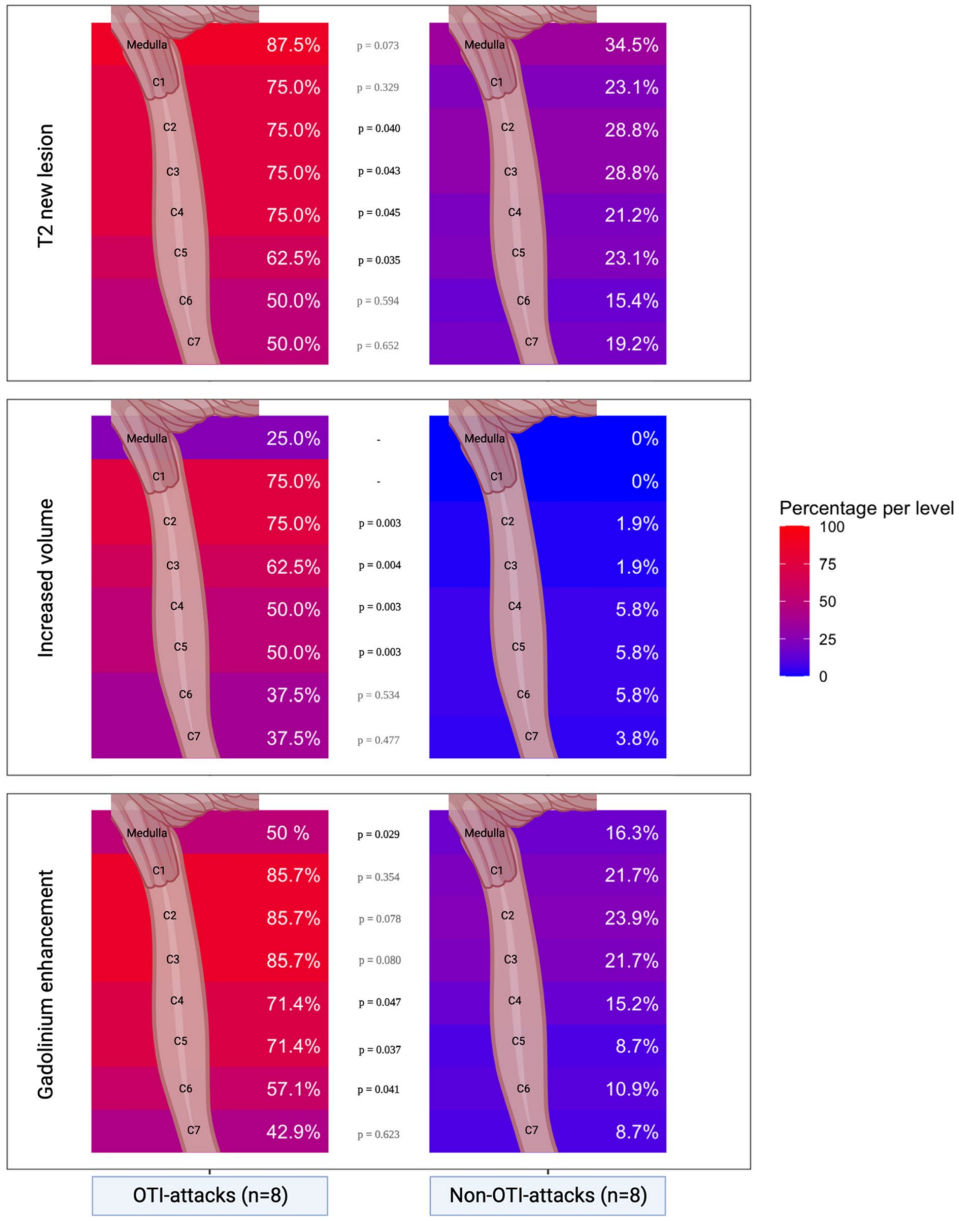
Medulla oblongata and Cervical Spinal Cord MRI characteristics of NMOSD attacks, comparing attack requiring OTI versus attacks not requiring OTI. *OTI* orotracheal intubation; percentages: number of attacks with radiological anomalies (new T2 lesion, gadolinium-enhancing lesion, increase volume)/number of attacks with available MRI, among OTI and non-OTI-attacks. *P*-value is estimated with binomial generalized linear mixed model, with a random intercept for patient ID. Due to separation issues, *p*-value has not been estimated for some variables

Spinal MRI was performed in 61 (81.3%) attacks and brain MRI in 52 (69.3%) attacks. Almost all OTI-attacks had extensive lesions in the upper cervical spinal cord. Only one OTI-attack had an isolated lesion in the medulla oblongata, with no involvement in the cervical spine. Brain and spinal MRI in OTI-attacks are shown in Fig. 1. In OTI-attacks, compared to non-OTI-attacks, new T2 lesions were more frequently observed in the cervical spinal cord at C2, C3, C4, and C5 levels, while gadolinium enhancement was more frequently observed at C4 and C5 levels, results are summarized in Table 2. As shown in Fig. 2, the MRI feature most strongly associated with OTI was upper cervical spinal oedema, which was present in 75% of OTI-attack MRI cases at the C1 and C2 levels, and 62.5% at the C3 level. In contrast, it was almost absent in non-OTI-attacks, with 0% of non-OTI-attack MRI cases at the C1 and C2 levels and only 1.9% at the C3 level.

**Table 2:** MRI characteristics of NMOSD attacks requiring orotracheal intubation versus attacks not requiring orotracheal intubation

	New T2 lesion			Gadolinium enhancing lesion		
	OTI-attacks	Non-OTI-attacks	*P*-value	OTI-attacks	Non-OTI-attacks	*P*-value
Patients with brain MRI—gadolinium injection performed during attack	7 (87.5)	45 (69.2)		7 (87.5)	39 (60.0)	
Brain location						
Medulla oblongata^a^	7 (87.5)	19 (34.5)	0.073	4 (50)	9 (16.3)	0.029
Pons	1 (14.3)	5 (11.1)	0.936	1 (14.3)	2 (5.1)	0.822
Midbrain	0 (0)	3 (6.7)	–	0 (0)	1 (2.6)	–
Diencephalon	0 (0)	4 (8.9)	–	1 (14.3)	3 (7.7)	0.883
Corpus callosum	1 (14.3)	2 (4.4)	0.954	1 (14.3)	2 (5.1)	0.963
Hemispheric	2 (28.6)	12 (26.7)	0.986	1 (14.3)	6 (15.4)	0.936
Optic nerve or chiasma	1 (14.3)	7 (15.6)	0.990	1 (14.3)	5 (12.8)	0.958
None	2 (28.6)	13 (28.9)	0.972	5 (71.4)	24 (61.5)	0.907
Patients with spinal MRI—gadolinium injection performed during attack	8 (100)	52 (80.0)		7 (87.5)	46 (70.7)	
Spinal cord level						
C1	6 (75)	12 (23.1)	0.329	6 (85.7)	10 (21.7)	0.354
C2	6 (75)	15 (28.8)	0.040	6 (85.7)	11 (23.9)	0.078
C3	6 (75)	15 (28.8)	0.043	6 (85.7)	10 (21.7)	0.080
C4	6 (75)	11 (21.2)	0.045	5 (71.4)	7 (15.2)	0.047
C5	5 (62.5)	12 (23.1)	0.035	5 (71.4)	4 (8.7)	0.037
C6	4 (50)	8 (15.4)	0.594	4 (57.1)	5 (10.9)	0.041
C7	4 (50)	10 (19.2)	0.652	3 (42.9)	4 (8.7)	0.623
Thoracolumbar	4 (50.0)	22 (42.3)	0.874	2 (25.0)	14 (26.9)	0.877
None	1 (12.5)	10 (19.2)	0.467	1 (14.3)	17 (36.9)	0.464

Results are expressed in median (interquartile range) or *N* (%) unless otherwise specified

*OTI* orotracheal intubation, *MRI* magnetic resonance imaging

*P*-value is estimated with binomial generalized linear mixed model, with a random intercept for patient ID. Due to separation issues, *p*-value has not been estimated for some variables

^a^Medulla oblongata T2 new lesion or gadolinium enhancement was assessed through radiological analysis of brain or spinal MRI

Spinal MRI performed during the two attacks requiring only non-invasive ventilation showed marked global spinal atrophy (data not shown).

### Treatment and prognosis of OTI versus non-OTI-attacks

**Fig. 3:**
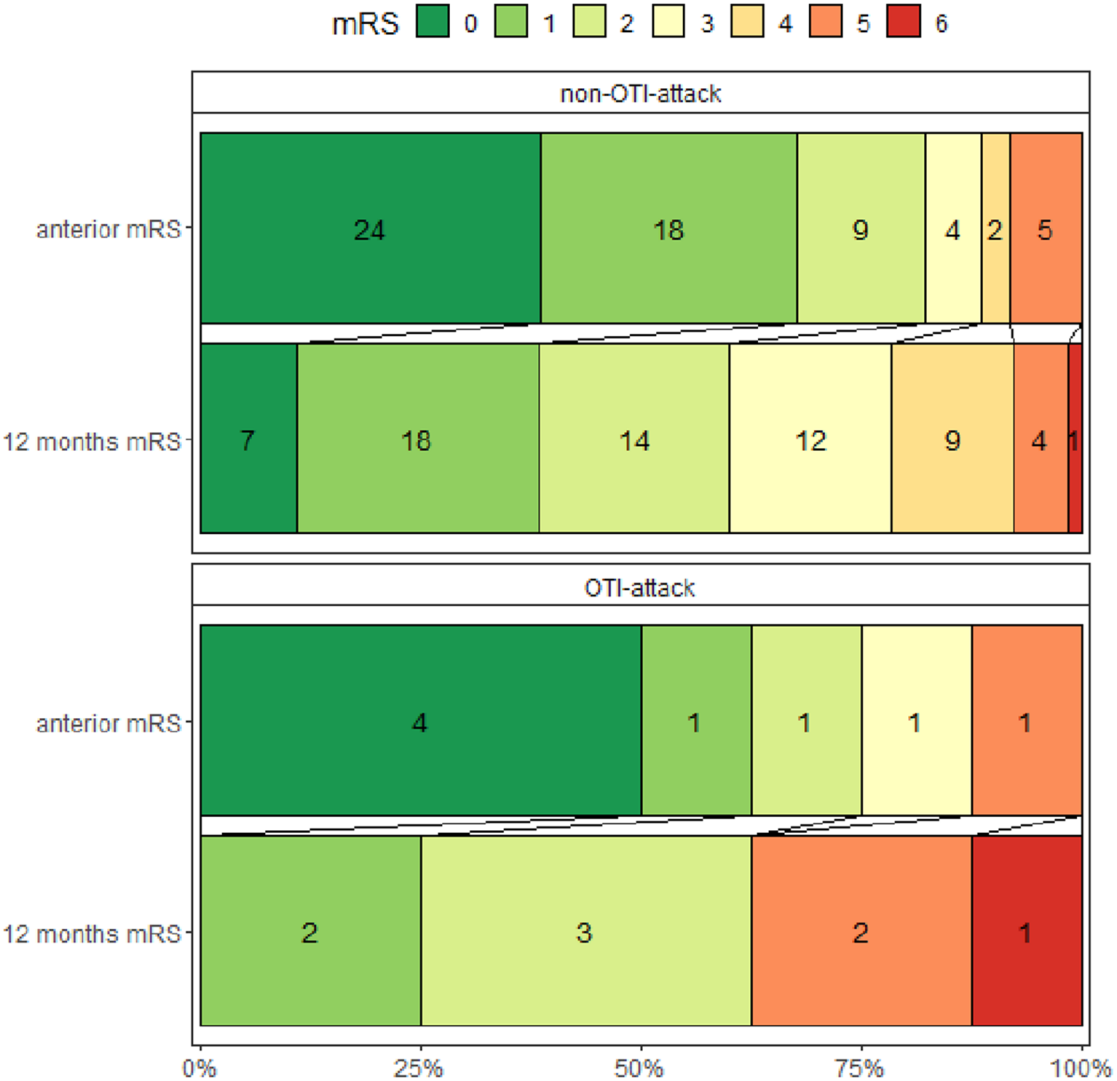
Change in modified rankin scale from Pre-Attack to 1 Year Post-Attack: OTI vs. Non-OTI-attacks. *OTI* orotracheal intubation, *mRS* modified rankin scale, *mRS 0* no residual symptoms, *mRS 1* no significant disability; able to carry out all pre-stroke activities, *mRS 2* slight disability; unable to carry out all pre-stroke activities but able to look after self without daily help, *mRS 3* moderate disability, requiring some external help but able to walk without the assistance of another individual, *mRS 4* moderately severe disability, unable to walk or attend to bodily functions without assistance of another individual, *mRS 5* severe disability, bedridden, incontinent, requires continuous care, *mRS 6* death

Treatment and prognosis of OTI and non-OTI-attacks are summarized in Table 3. The median delay from attack’s first symptoms to hospitalization was 18 (7.5;32.5) days for OTI-attacks and 10.5 (3.8;21) days for non-OTI-attacks (*p* = 0.779). Eight OTI-attacks (100%) and 27 (41.5%) non-OTI-attacks were treated with TPE. The median delay from hospitalization to OTI was 1 (0;4.25) day. The median duration of OTI was 27 days (minimum 4; maximum 96). Three OTI-attacks (37.5%) had ventilation-associated pneumonia. Three OTI-attacks (37.5%) required temporary tracheostomy. One patient requiring OTI (12.5%) died during the attack. This patient was 86 years old and died due to ventilator-associated pneumonia, which was secondary to the attack. All survivors of OTI-attacks remained independent of mechanical ventilation at 3-, 6-, and 12-months post-attack. Only one survivor of an OTI-attack required temporary nocturnal non-invasive ventilation for 1 month after the attack. A recurrent attack during the following year were observed in 1 (12.5%) OTI versus 28 (43.1%) non-OTI (*p* = 0.624). Two OTI-attacks (25.0%) had an unfavorable outcome at 12 months, compared to 15 (24.2%) non-OTI-attacks (*p* = 0.468). Of the two OTI-attacks with unfavorable outcome, one resulted in death, and the other in severe persistent neurological disability characterized by paraplegia and upper limb paresis, with the patient eventually becoming bedridden. One year after the attack, five patients with OTI-attacks had a modified Rankin Score ≤ 2, four were able to walk at least 500 m unassisted (EDSS < 4) and two were actively employed. The change in mRS from Pre-Attack to 1 year post-attack for OTI vs. non-OTI-attacks is summarized in Fig. 3.

**Table 3:** Treatment and prognosis of attacks requiring orotracheal intubation versus attacks not requiring orotracheal intubation

Hospitalization duration, days	21 (7–48)	74 (55–98)	21 (7–36)	0.061
Delay from attack first symptoms to hospitalization, days	11.5 (4–21.2)	18 (7.5–32.5)	10.5 (3.8–21)	0.779
ICU hospitalization: yes	28 (38.4)	8 (100)	20 (27.4)	-
If yes: ICU duration, days, median (P25;P75) (*N* attacks with ICU hospitalization)	25 (12;40) (28)	44.5 (40;57) (8)	17 (7;28) (20)	0. 094
If yes: SAPS II, median (P25;P75) (*N* attacks with ICU hospitalization)	13 (6;18.25) (28)	19 (17;19) (8)	13 (6;13) (20)	0.891
Intubation duration, days	–	27.00 [17.00, 41.50]	–	–
Aspiration pneumonia	–	3 (37.5)	–	–
Ventilator-associated pneumonia	–	5 (62.5)	–	–
Tracheostomy	–	2 (37.5)	–	–
Medical treatment				
Total dose of corticosteroids (grams)	7.5 (5;10)	10 (8;10)	7 (5;10)	0.677
Delay from first symptoms to IV corticosteroids, days	14 (7–32)	24.5 (13–58.5)	13 (6–30)	0.701
TPE (yes or no)	35 (47.9)	8 (100)	27 (41.5)	–
Number of TPE	6 (5–8)	7.5 (6.3–10)	6 (5–8)	0.911
Delay from first symptoms to TPE, days	21.5 (12.5–44)	28 (16.5–82)	20 (9.3–38)	0.599
Outcomes				
Death during hospitalization	1 (1.3)	1 (12.5)	0 (0.0)	–
EDSS 3 months after attack onset, median (IQR) (NA)	4.0 (2.5–7.0) (13)	7.0 (6.0–9.0) (0)	3.5 (2.5–6.5) (13)	0. 402
EDSS 6 months after attack onset	3.5 (2.0–7.0) (14)	5.5 (2.5–9.0) (0)	3.5 (2.5–6.5) (14)	0.574
EDSS 12 months after attack onset (0)	3.5 (2.0–6.5) (0)	5.0 (2.0–9.0) (0)	3.5 (2.0–6.0) (0)	0.524
EDSS < 4, 12 months after attack	38 (52.1)	4 (50.0)	34 (52.3)	0.476
Recurrent attack during the following year	29 (39.7)	1 (12.5)	28 (43.1)	0.624
Patient working 12 months after attack	22 (33.8)	2 (28.5)	20 (34.5)	0.028
Modified Rankin scale 12 months after the attack	2 [1, 3]	2 [1, 3]	2 [1, 3.5]	0.549
Unfavorable attack recovery^a^ at 12 months	17 (24.3)	2 (25.0)	15 (24.2) (3)	0.468

Results are expressed in median (interquartile range) or *N* (%) unless otherwise specified

*NA* number of observations non available, *OTI* orotracheal intubation, *TPE* therapeutic plasma exchange, *EDSS* expanded disability severity score

^a^Unfavorable attack recovery = defined as modified Rankin score (mRS) > 2 at 3 months for patients with initial mRS ≤ 2 or an increase of ≥ 1 point in mRS for patients with initial mRS > 2

### Assessing OTI during an attack as a predictor of attack recovery

OTI was not significantly associated with unfavorable outcome at 12 months either in univariate analysis (OR for unfavorable outcome 0.94 [0.11;8.28]) or in multivariate analysis, adjusting for age, EDSS at admission, and treatment with TPE (OR for unfavorable outcome 0.47 [0;3.22]), results are summarized in Table 4.

**Table 4:** Orotracheal intubation during an attack as a predictor of attack recovery

	Univariate odd ratio (IC 95%)	Multivariable odd ratio (IC 95%)
Age	1.1 (0.99;1.21)	1.11 (1;1.23)
Gender:woman	6.04 (0.46;79.72)	
Attack occurring in the first 6 months of NMOSD-AQP4 disease	1.48 (0.49;4.44)	
EDSS at admission	1.63 (1.11;2.38)	1.68 (0.92;3.46)
Previous DMT	0.39 (0.1;1.55)	
Attack requiring orotracheal intubation	0.94 (0.11;8.28)	0.47 (0;3.22)
Attack treated with TPE	5.9 (1.21;28.83)	3.21 (0.45;56.2)

Unfavorable attack recovery = defined as modified Rankin score (mRS) > 2 at 12 months for patients with initial mRS ≤ 2 or an increase of ≥ 1 point in mRS for patients with initial mRS > 2

Variables included in the multivariable logistic regression were OTI and variables associated with unfavorable attack recovery in the univariate analysis. Odd ratios were estimated with binomial generalized linear mixed model, with a random intercept for patient ID

*EDSS* expanded disability severity score, *DMT* disease modifying therapy, *TPE* therapeutic plasma exchange

### Characteristics of attacks requiring ICU admission

Twenty-eight attacks (38.4%) required admission to ICU, and the results of which are summarized in Additional file 1: Table S1. The reasons for ICU admission (not mutually exclusive) were as follows: symptoms of respiratory insufficiency (10 [35.7%]), swallowing difficulties (5 [17.9%]), suspected superimposed infection (4 [14.3%], all originating from the respiratory tract), altered consciousness (3 [10.7%]), and/or urgent need for therapeutic plasma exchange (8 [28.6%]). The SAPS II score on admission to the ICU was 13.00 (6.00, 18.25).

## Discussion

In our study, NMOSD-AQP4-IgG+ OTI-attacks were rare events occurring in untreated patients and almost exclusively in the first 6 months after disease onset. On admission, high acute neurological disability, upper motor arm paresis and respiratory signs were early warning signs of respiratory failure. All OTI-attacks presented with lesions of the cervical spine or medulla oblongata, mainly associated with cervical oedema. One OTI-attack resulted in the death of an 86-year-old patient, but the prognosis for other OTI-attacks be favorable, as a significant number of the patients were ambulatory 1 year after OTI-attacks.

NMOSD-AQP4-IgG+ is a rare disease (prevalence ranging from 3.9 to 10/100,000 [8]), and the occurrence of OTI during attacks is even rarer: only 8 cases in a decade at our center. However, these attacks accounted for 11% of all hospitalized encephalic or spinal attacks during this period, a proportion higher than that reported previously [9, 14, 15]. This discrepancy may be due to the exclusion of isolated cases of optic neuritis in our study or to a center effect due to the existence of a referral center specialized in neurological intensive care at the Pitié-Salpêtrière hospital, which admits severe patients from all over the France.

None of the patients experiencing OTI-attack was previously treated with active DMT. The absence of DMT is a major risk factor for NMOSD-AQP4-IgG+ attack [6], but our results suggest that it could also contribute to attack severity with respiratory failure. Our results are consistent with a recent work from Mayo-clinic [15] in which only one from eleven NMOSD-AQP4-IgG+ with OTI-attacks had a known previous standard NMOSD-AQP4-IgG+ DMT. These results underline the need for early diagnosis of NMOSD-AQP4-IgG+ using the IPND 2015 criteria [5], to promptly start DMT. In addition, the first neurological symptoms of OTI-attacks began up to 94 days before OTI, suggesting a likely early treatment window to prevent respiratory failure. High acute neurological disability, upper limb paresis and dyspnea were often the first clinical features of OTI-attacks. These clinical signs, if present at the time of admission for an NMOSD-AQP4-IgG+ attack, warrant close medical monitoring and may justify monitoring in intensive care unit.

The main radiological feature of OTI-attacks was the presence of cervical spine oedema. Only one patient had isolated lesion of medulla oblongata. Our results suggest that in NMOSD-AQP4-IgG+, respiratory failure may be related to the both high cervical motor control of the respiratory muscles (C1 to C4) and respiratory centers in the medulla oblongata [16–18].

In our experience, only two attacks required non-invasive ventilation in a single patient with long-standing tetraparesis, suggesting probable underlying chronic respiratory insufficiency. The spinal cord MRI showed minimal new lesions in the cervical region and no oedema; but atrophy of the spinal cord. This pattern differed markedly from OTI-attacks.

Historical cohorts (medical records from 1950 to 1993) described a 93% risk of death among NMOSD-attacks with acute respiratory failure [9], which contrasts with our experience of only one death in 8 OTI-attacks. This huge difference could be explained by the specific medical care in the acute phase: OTI, ICU monitoring, and high doses of methylprednisolone associated with TPE. Furthermore, the functional prognosis of OTI-attacks may also be favorable, as all survivors were free of ventilatory support and 4 patients with OTI-attacks were walking unassisted 1 year after OTI-attack. This justifies the need for careful medical monitoring and aggressive treatment in cases of severe NMOSD-AQP4+ attacks, including intubation (OTI) if necessary, without any compromising of care.

The strengths of our study are the comparative group of patients with encephalic or spinal NMOSD-AQP4-IgG+ attacks, the comparative analysis of radiological findings, and the homogenous attack management strategy over the study period. In addition, unlike other studies [15], we focused on the risk factor and prognosis for OTI-attacks, which have different indications compared to non-invasive ventilation [19].

Our study has some limitations. Firstly, the Pitié-Salpêtrière hospital group is an expert center for inflammatory diseases with an ICU specialized in these conditions, which may introduce a referral bias for the more severe cases, limiting the generalization of our findings. The clinical approach at our center is to admit NMOSD-AQP4+ patients to the ICU in the presence of impending or existing respiratory failure, or to initiate TPE promptly. However, harmonization of criteria for ICU admission and intubation, as well as their routine collection in the NOMADMUS cohort, would allow multicenter studies to be conducted. Our study may also have had limited statistical power to analyze the differences between OTI and non-OTI-attacks due to a small number of OTI-attacks. For example, the lack of a significant association between OTI and attack recovery does not necessarily mean that OTI is not associated with the risk of long-term sequelae.

## Conclusion

NMOSD-AQP4-IgG+ attacks rarely lead to respiratory failure. OTI-attacks occur early in the course of the disease and in untreated patients. MRI scans during OTI-attacks show extensive edematous lesions in the upper cervical spinal cord and the medulla oblongata. This threat contrasts with the potential for a good prognosis of NMOSD OTI-attacks. Therefore, the severity of acute neurological dysfunction should not be used as a basis for limiting or restricting care. Instead, these attacks should be managed in expert centers with an intensive care unit.

### Supplementary Information

**Additional file 1: Table S1.** Clinical characteristics of attacks hospitalized in ICU versus attacks not hospitalized in ICU.

## Data Availability

Caroline Papeix and Edouard Januel had full access to all the data in the study and take responsibility for the integrity of the data and the accuracy of the data analysis.
