# Supplementary material for: Risk factors and prognosis of orotracheal intubation in aquaporin-4-IgG neuromyelitis optica spectrum disorder attacks
Source: Ann Intensive Care. 2024 Jan 8;14:4. doi: 10.1186/s13613-023-01213-x (PMC10772133; doi:10.1186/s13613-023-01213-x)
Supplement: Supplementary file 1 — Additional file 1: Table S1. Clinical characteristics of attacks hospitalized in ICU versus attacks not hospitalized in ICU. [file 13613_2023_1213_MOESM1_ESM.docx]

**Table S1: Clinical characteristics of attacks hospitalized in ICU versus attacks not hospitalized in ICU**

|  | All attacks,  N=73 | ICU attacks, N=28 (38.4%) | Non-ICU attacks, N=45 (61.6%) | P value |
| --- | --- | --- | --- | --- |
| Gender: female/male | 64 (87.7) | 23 (82.1) | 41 (91.1) | 0.399 |
| Place of birth |  |  |  | - |
| France – metropolitan area | 34 (46.6) | 8 (28.6) | 26 (57.8) |  |
| France – West indies – Guyana | 6 ( 8.2) | 5 (17.9) | 1 ( 2.2) |  |
| Asia | 6 ( 8.2) | 1 ( 3.6) | 5 (11.1) |  |
| North Africa | 6 ( 8.2) | 2 ( 7.1) | 4 ( 8.9) |  |
| Sub-Saharan Africa | 20 (27.4) | 11 (39.3) | 9 (20.0) |  |
| Middle East | 1 ( 1.4) | 1 ( 3.6) | 0 ( 0.0) |  |
| Age at start of the disease, years, median (IQR) | 33.20 [21.30, 46.90] | 42.25 [32.90, 50.60] | 31.00 [17.90, 39.80] | 0.177 |
| Age, years | 41 [31-53] | 46 [34- 52] | 39 [27, 53] | 0.298 |
| First attack of NMOSD-AQP4 disease | 22 (30.1) | 11 (39.3) | 11 (24.4) | 0.251 |
| Attack occurring in the first six months of NMOSD-AQP4 disease | 29 (39.7) | 15 (53.6) | 14 (31.1) | 0.257 |
| Any active NMOSD disease modifying therapy (DMT)* | 36 (49.3) | 6 (21.4) | 30 (66.7) | 0.006 |
| Anterior rankin |  |  |  | 0.855 |
| EDSS before attack | 2 [0-3.5] | 1 [0-4] | 2 [0-3.5] | 0.775 |
| EDSS at admission | 6 [3.5-8] | 7.25 [4-8] | 5 [3-7] | 0.073 |
| Lower limb paresis | 46 (63.0) | 21 (75.0) | 25 (55.6) | 0.213 |
| Upper limb paresis | 25 (34.2) | 17 (60.7) | 8 (17.8) | <0.001 |
| Reason for admission in ICU, N (% of admitted in ICU)** |  |  |  |  |
| Respiratory | - | 10 (35.7) | - | - |
| Swallowing disorder | - | 5 (17.9) | - | - |
| Suspected infection | - | 4 (14.3) | - | - |
| Altered consciousness | - | 3 (10.7) | - | - |
| Admission for TPE | - | 8 (28.6) | - | - |
| SAPS II score | - | 13 [6-18] | - | - |
| Mechanical ventilation in ICU, N (% of admitted in ICU) | - | 10 (35.7) | - | - |
| If Yes : OTI | - | 8 (28.6) | - | - |
| If Yes : Non invasive ventilation without OTI | - | 2 (7.1) | - | - |

Results are expressed in median (interquartile range) or N (%) unless otherwise specified; ICU: intensive care unit; *active DMT was defined if it has been received for at least 3 months before the attack; Suspected infection: all were respiratory tract infections; EDSS : expanded disability severity score ; ** Reason for admission in ICU are not mutually exclusive; TPE: Therapeutic Plasma Exchange; SAPS II : Simplified Acute Physiology Score II ; OTI : orotracheal intubation ; P-value are estimated with binomial generalized linear mixed model, with a random intercept for patient ID. Due to separation issues, p value has not been estimated for some variables
